# Naturally derived injectable hydrogels with ROS-scavenging property to protect transplanted stem cell bioactivity for osteoarthritic cartilage repair

**DOI:** 10.3389/fbioe.2022.1109074

**Published:** 2023-01-04

**Authors:** Haobo Li, Dong Xiang, Chongcheng Gong, Xiaomin Wang, Lin Liu

**Affiliations:** ^1^ Department of Orthopaedics and Traumatology, Shanghai East Hospital, School of Medicine, Tongji University, Shanghai, China; ^2^ Department of Orthopaedics, Shanghai Changzheng Hospital, Naval Medical University, Shanghai, China

**Keywords:** hydrogel, anti-oxidant, stem cell, cartilage repair, osteoarthritis

## Abstract

Intra-articular injection of adipose mesenchymal stem cells (ADSCs) is a potential alternative to the treatment of osteoarthritis (OA) and has aroused great interest of clinical researchers. However, the hostile microenvironment in the joint cavity, characterized by reactive oxygen species (ROS) accumulation and excessive inflammation, disturbs the bioactivity of the transplanted stem cells. The (-)-epigallocatechin-3-O-gallate (EGCG), a green tea catechin, has attracted the researchers’ attention owing to its powerful ROS-scavenging and antioxidant properties. In this study, to avoid rapid degradation and/or depletion of EGCG, we prepare a long-lasting injectable hydrogel by EGCG and hyaluronic acid (HA). The naturally derived hydrogels with excellent biocompatibility and durable retention time can capture the redundant ROS continuously and efficiently, thus protecting ADSCs from ROS-mediated death and bioactivity inhibition, including cell survival, proliferation and chondrogenic differentiation. Intra-articular injection of this ADSCs loaded hydrogel significantly induced synovial macrophages polarization to M2 phenotype, decreased pro-inflammatory cytokines (e.g., IL-1β, MMP-13, and TNF-α) expression, promoted cartilage matrix formation, and repaired cartilage destruction in OA. This stem cell-protected hydrogel delivery strategy showed superior efficacy than ADSCs delivering or EGCG-HA injection singly, which providing a potential alternative strategy for OA management.

## Introduction

Osteoarthritis (OA) is the most common chronic joint disease in orthopedics, characterized by articular cartilage degeneration, reactive hyperplasia of articular border and subchondral bone. OA has a complex disease process associated with synovium, articular cartilage, subchondral bone, ligament, joint capsule and muscles around joints (Huang et al., 2022). With the accelerating of population aging, the population suffering from OA has reached 200 million, which results in huge living obstacles and heavy economic burden to the society (Li Y. et al., 2022; Zhao et al., 2022). Over the past decades, intra-articular injection of various stem cells has emerged as an advanced therapy to delay the pathological progress of OA. Among them, some mesenchymal stem cells (MSCs), including adipose mesenchymal stem cells (ADSCs) and bone marrow mesenchymal stem cells (BMSCs), have become the most preferred therapeutic cells for treating OA owing to their extensive sources, accessibility, multi-directional differentiation, and the enviable paracrine effects (Molnar et al., 2022; Shoukrie et al., 2022; Zeng et al., 2022). Intra-articular injection of MSCs is well-proven as an alternative treatment to repair damaged cartilage, delay the pathological progress of OA, as well as relieve pain (Csaki et al., 2008; Maumus et al., 2011; Yin B. et al., 2022; Lin et al., 2022). However, the stem cells delivered to the articular cavity are seriously affected by the harsh microenvironment of OA. As a result, the stem cells are difficult to give full play to their multiple functions, thus limiting the wide application of this strategy.

The pathological microenvironment of OA is multifaceted and involves the whole joint (Kang et al., 2022). Recently, growing evidences suggested that accumulated reactive oxygen species (ROS) and oxidative stress show an increasingly important role in OA microenvironment (Ahmad et al., 2020). ROS participate in various biological processes and are closely associated with the aggravation of OA. The accumulated ROS will activate a series of signal pathways, subsequent lead to chondrocytes catabolism and apoptosis, matrix metalloproteases (MMPs) overexpression, extracellular matrix (ECM) degradation, and excessive inflammation, thus causing various pathological changes of OA (Li et al., 2017; Khan et al., 2018; Stocco et al., 2019). The balances of ROS production and deletion are greatly related to the occurrence, development, and progress of OA. Previously, Kim et al*.* (2019) developed manganese ferrite and ceria co-decorated nanoparticles that can scavenge ROS and produce O_2_ synergistically. The functional nanoparticles alleviated hypoxia and excessive inflammation, induced inflammatory M1 macrophages polarization toward anti-inflammatory M2 phenotype, thus remodeling the pathological features of the arthritic joint. Therefore, removing excessive ROS to reshape the pathological microenvironment of OA to enable transplanted stem cells to give full play to their functions, may be an alternative therapy for OA treatment.

The hydrogels composed of hydrophilic three-dimensional (3D) network structure are widely used to deliver bioactive substances and stem cells to promote tissue repair and regeneration due to their fascinating properties (Li et al., 2021; Li et al., 2022b). In this study, we prepared a functional hydrogel, synthesized by thiolated hyaluronic acid (HA) and (-)-epigallocatechin-3-O-gallate (EGCG) quinone, as a protective carrier of stem cells for intra-articular injection to treat OA. As the main ingredient of green tea catechins, EGCG has attracted much interest in the field of biomedicine due to its enviable ROS-scavenging capacity, anti-oxidation, and anti-inflammatory performances (Yang et al., 2022; Zhu et al., 2022). By nucleophilic addition reaction of thiolated HA and EGCG quinone, the HA-EGCG hydrogel was synthesized and showed excellent ROS-scavenging ability and hyaluronidase (HAase)-inhibitory activity (Liu et al., 2017). Herein, we investigated the degradation resistance, ROS-scavenging ability, and protective effect on ADSCs chondrogenic differentiation of HA-EGCG hydrogel under oxidative stress state, to demonstrate its potential as a cytoprotective carrier in the management of OA.

## Materials and methods

### Materials

EGCG, HAase, xanthine, 2′, 7′-dichlorodihydrofluorescein diacetate (DCFH-DA), and streptomycin–penicillin were supplied by Sigma-Aldrich (St. Louis, MO, United States). Xanthine oxidase from buttermilk was provided by Oriental Yeast Co. (Osaka, Japan). Thiolated HA was provided by Qiyue Biotechnology Co., Ltd (Xi’an, China). Commercial HA was supplied by Boshilun Furida Pharmaceutical Co., Ltd (Shandong, China). Hydrogen peroxide (H_2_O_2_) and hematoxylin eosin (H&E) stain, Alcian blue stain, and Safranin O stain were purchased from Thermo Fisher Scientific (Waltham, MA, United States). Horseradish peroxidase (HRP) and fetal bovine serum (FBS) were provided by Yuanye Biotechnology Co., Ltd (Shanghai, China). Low glucose Dulbecco modified Eagle medium (DMEM) and 0.25% trypsin EDTA were obtained from HyClone (Logan, Utah, United States). Mice ADSCs and chondrogenic differentiation induction medium of ADSCs were supplied by Procell (Wuhan, China). Cell Counting Kit-8 (CCK-8) assay was purchased from Dojindo (Japan) and Calcein-AM/Propidium Iodide (PI) was supplied by Beyotime Biotechnology (Shanghai, China). Phosphate buffer saline (PBS) and 4% paraformaldehyde were obtained from Solarbio (Beijing, China). The enzyme-linked immunosorbent assay (ELISA) kits of interleukin-1 beta (IL-1β), matrix metalloproteinase-13 (MMP-13), tumor necrosis factor-alpha (TNF-α) were supplied by Hengyuan Biotechnology Co., Ltd (Shanghai, China). Eastep Super Total RNA Extraction Kit was supplied by Promega (Shanghai, China) and Perfect Real Time RT reagent kit was obtained from Takara Bio (Dalian, China). Primary antibodies were provided by Abcam (Cambridge, United Kingdom), and secondaries antibodies were supplied by Jackson ImmunoResearch Laboratories (West Grove, PA, United States). All other chemicals and reagents were of analytical grade.

### HA-EGCG hydrogel preparation and characterization

The HA-EGCG conjugate was synthesized by coupling EGCG to thiolated HA derivatives as described previously (Liu et al., 2017). In brief, we dissolved thiolated HA (1 g) in the 140 ml PBS (pH = 7.4) under nitrogen atmosphere, then added drop by drop to 60 ml PBS (pH = 7.4) containing excess EGCG, and stirred continuously. Adjust the pH of the solution to 7.4 by dropping NaOH, and react at 25°C for 3 h, then adjust the pH to 6. The obtained solution was dialyzed under nitrogen atmosphere, and then the purified solution was freeze-dried to acquire HA-EGCG conjugate.

HA-EGCG conjugate was dissolved in deionized water at 25 °C to acquire the HA-EGCG solution (11.5 mg/ml) firstly. To obtain the HA-EGCG hydrogels, 7.8 ml HA-EGCG solution was merged with 150 μL of HRP solution, 150 μL of H_2_O_2_ solution, and 900 μL PBS (pH = 7.4) in a tube for 24 h. Rheological measurements were performed with a RH-20 rheometer (Bosin Tech, China) at room temperature. The viscosity of the hydrogels was tested by a viscometer (NDJ-5S, Shanghai, China).

### Degradation of HA-EGCG hydrogel

To evaluate the degradation of hydrogel, 1 g HA-EGCG hydrogel was placed in glass bottles and immersed with 5 ml PBS or HAase (15 units/ml) contained PBS at 37°C. During the whole degradation process, HAase was supplemented every day. At the predetermined time intervals, the residual hydrogels were collected and weighed. The residual ratio of hydrogels was determined by following formula: residual hydrogel ratio (%) = W_t_/W_0_ × 100%. The Wt is the hydrated mass at the measured time point and W_0_ is the initial weight of the hydrogel. Commercialized HA for intra-articular injection clinically was used as the control agent.

### Biocompatibility of HA-EGCG hydrogel

ADSCs were seeded in a 48-well plate with density of 1×10^4^/well. The plates pre-coated with 100 μL HA-EGCG hydrogel was used to investigate the biocompatibility of the material. The cells were incubated in the incubator for 2 days, and Calcein-AM/PI was performed according to the manufacturer’s protocols. Briefly, 5 μM Calcein-AM solution was added into the samples for 15 min, subsequent 5 μM PI solution was transferred to the wells for 5 min, which conducted at room temperature and away from light. The pictures were observed by a confocal microscope (Olympus Fluoview FV1000, Japan). In addition, the proliferation of ADSCs cultured in different substrates were detected by CCK-8 test at predetermined time points. Briefly, the culture medium was removed and 10% CCK-8 solution was added to each well. The samples were incubated at 37 °C for 2 h and then transferred to a 96-well plate for absorbance detection by a microplate reader (Varioskan LUX, Thermo Fisher Scientific, MA, United States) at 450 nm.

### ROS-scavenging capacity

The H_2_O_2_ depletion ability of the HA-EGCG hydrogel was monitored by mixing H_2_O_2_ (1 mM, 1 ml) and hydrogel (1 ml) in PBS at 37 °C as described previously (Li et al., 2022a). After designated times, the supernatant (100 µL) was collected and added 200 µL of Ti(SO_4_)_2_ solution. After incubation for 30 min, the H_2_O_2_ concentration was determined by testing the absorbance of samples at 405 nm by a microplate reader.

### Intracellular ROS (H_2_O_2_) depletion and cell viability protection

The intracellular H_2_O_2_ scavenge capacity of HA-EGCG hydrogel was evaluated by a H_2_O_2_ probe DCFH-DA. Specifically, ADSCs were seeded in 48-well plates pre-coated with 100 μL HA or 100 μL HA-EGCG hydrogel at the density of 1×10^4^/well under the oxidative environment (100 µM H_2_O_2_ in the culture medium). For comparison, ADSCs were also incubated with 100 μL PBS in the presence or absence of H_2_O_2_, respectively. After incubating for 2 days, DCFH-DA agent was transferred to various groups for 20 min at room temperature. The fluorescence intensity indicating the intracellular ROS level of the cells was observed by the confocal microscope. Furthermore, under the oxidative stress and the protection of HA-EGCG hydrogel, the cell viability was evaluated by a Calcein-AM/PI kit and observed with the confocal microscope.

### Real-time PCR analysis

ADSCs were seeded in 48-well plates pre-coated with 100 μL HA or 100 μL HA-EGCG hydrogel at the density of 1×10^4^/well under the oxidative environment as described above. After incubation with chondrogenic induction medium for 14 days, cell samples were collected for real-time PCR analysis. In brief, the samples were ground within TRIzol reagent to extract total mRNA. The concentration of acquired RNA was determined by Infinite 200 PRO NanoQuant Microplate Readers (TECAN). Then the RNA (1 μg) was used for cDNA synthesis. Real-time PCR was performed using 2× Fast SYBR Green Master Mix (Roche Diagnostics, Basel, Switzerland) and ABI StepOnePlus™ Real-Time RCR system (Applied Biosystems). The relative mRNAs expression was calculated by the 2^−ΔΔCt^ formula. The primer sequences of GAPDH, IL-1β, MMP-13, TNF-α, aggrecan (ACAN), type II collagen (COL-2), and Sex determining region Y box 9 (SOX-9), were listed in Table 1.

**TABLE 1:** Primer sequences of genes.

Gene	Oligonucleotide primers (5′-3′)
*GAPDH*	F: 5′- TCA GCT GCT GGG GAG TCA CA-3′
	R: 5′- CCT AAG CCC CTC CCC TTC TT-3′
*IL-1β*	F: 5′- TCT CTG GAC CCA AAG GAG GG-3′
	R: 5′- ACG GCT GCT TTC ACG GGT GA-3′
*MMP-13*	F: 5′- CCT GTG CTC CTG CCA TTT GG-3′
	R: 5′- GAA TGG GCA GCT CCA TGG CT-3′
*TNF-a*	F: 5′- AGC TGG TGG TGC CGA CAG AT-3′
	R: 5′- TGC GAT GCG GCT GAT GGT GT-3′
*ACAN*	F: 5′- CTG CCC CGA AAC ATC ACC GA-3′
	R: 5′-GTA AAG GGC TCC TCA GGC TC-3′
*COL-2*	F: 5′-TAG AGA GGT TTC CTG GGC CG-3′
	R: 5′-AGG AGG ACG CTG GAA CAG AG-3′
*SOX-9*	F: 5′-CAG TCC CAG CGA ACG CAC AT-3′
	R: 5′-TGC TGC TGC TGC TCG CTG TA-3′

### Alcian blue and safranine O staining

The chondrogenic differentiation potential of ADSCs under the oxidative environment with the protection of HA-EGCG hydrogel was evaluated by staining the samples with Alcian blue and Safranin O dyes according the manufacturer’s instructions. In brief, after culture in chondrogenic induction medium for 14 days, the cells were fixed by 4% paraformaldehyde for 5 min, followed by being stained with Alcian blue for 30 min at room temperature, and the remaining samples were incubated with Safranin O for 5 min according to the manufacturer’s instructions. Finally, the stained pictures were observed and photographed with a DSX 500 optical microscope (Olympus, Japan).

### OA model preparation and intra-articular administration

Sprague-Dawley rats (male, eight-week-old, ∼250 g) were used to prepared OA model according to classic surgical destabilization of the medial meniscus (DMM) as described previously (Shao et al., 2021). Briefly, the rats were anesthetized by 3% pentobarbital injected intraperitoneally at the dose of 0.2ml/100g, and then the joint cavity was opened layer by layer and the medial collateral ligament was transacted. Four weeks after molding, intra-articular injection of different therapeutic agents was implemented once. Specifically, therapeutic agents included 100 μl PBS as negative control, 100 μl PBS containing 2×10^6^ ADSCs, 100 μl HA-EGCG hydrogel, and 100 μl HA-EGCG hydrogel containing 2×10^6^ ADSCs, abbreviated as PBS, ADSCs, Gel, and ADSCs/Gel, respectively. At the sixth week, the animals in each group received another intra-articular injection of therapeutic agents. Twelve weeks after the first injection, the experimental animals were euthanized and the articular cartilages and surrounding synovial tissues were collected for subsequent investigations.

### Evaluation of inflammatory cytokines in synovium

Some inflammatory cytokines, including IL-1β, MMP-13, and TNF-α in the synovium tissues were evaluated by corresponding ELISA kits based on the manufacturer’s protocols. In brief, 500 mg synovium tissues were collected, uniformly cut, and transferred to protease inhibitor contained PBS (5 ml). The synovium samples were placed in a glass homogenizer and grinded fully on ice. Subsequently, the absorbance of various samples was determined by the microplate reader at 450 nm. The concentrations of IL-1β, MMP-13, and TNF-α were determined according to the standard curve.

### Histological sections preparation and staining

After the rats were sacrificed, the synovium and cartilage tissues were preserved and then fixed by 4% paraformaldehyde. The synovium tissues were embedded in paraffin for subsequent conventional histological processing and ∼5 μm thickness slices were prepared. For cartilage tissues, the samples were decalcified with 0.5 M EDTA solution for 1 month first, and then prepared ∼5 μm thickness slices. And then, the sections were incubated with H&E dye according to the instructions for subsequent histological observation.

### Immunofluorescence staining

To study macrophages polarization, the sections of synovium tissues were stained with CD68 plus iNOS and CD68 plus CD163. The cartilage tissues were marked by ACAN, COL-2, and SOX-9. In brief, the sections were blocked with 3% BSA, and 0.2% Triton X-100 for 1 h. The synovium samples were incubated with primary antibodies, anti-CD68 antibody (1:100) plus anti-iNOS antibody (1:200), and anti-CD68 antibody (1:100) plus anti-CD163 antibody (1:150) at 4°C overnight. The sections of cartilage tissues were incubated with primary antibodies, anti-COL-2 antibody (1:200) plus anti-SOX-9 antibody (1:150), and anti-ACAN antibody (1:150). After washing by PBS for three times, the sections were incubated with secondary antibodies Cy3-conjugated goat anti-rabbit (1:500) and goat anti-mouse IgG DyLight 488-conjugated (1:600) for 1 h at room temperature. At last, the nucleus was stained with DAPI for 3 min, the images were observed and photographed by a confocal microscope. The relative fluorescence intensity of images was quantitatively analyzed with ImageJ software.

### Statistical analysis

All results were presented as mean ± standard deviation. Statistical analysis was performed by SPSS (version 22.0, Chicago, IL, United States) and the difference between values was analyzed using Student’s unpaired *t*-test. All experiments repeated at least 3 times independently.

## Results and discussions

### HA-EGCG hydrogel preparation and characterization

**FIGURE 1:**
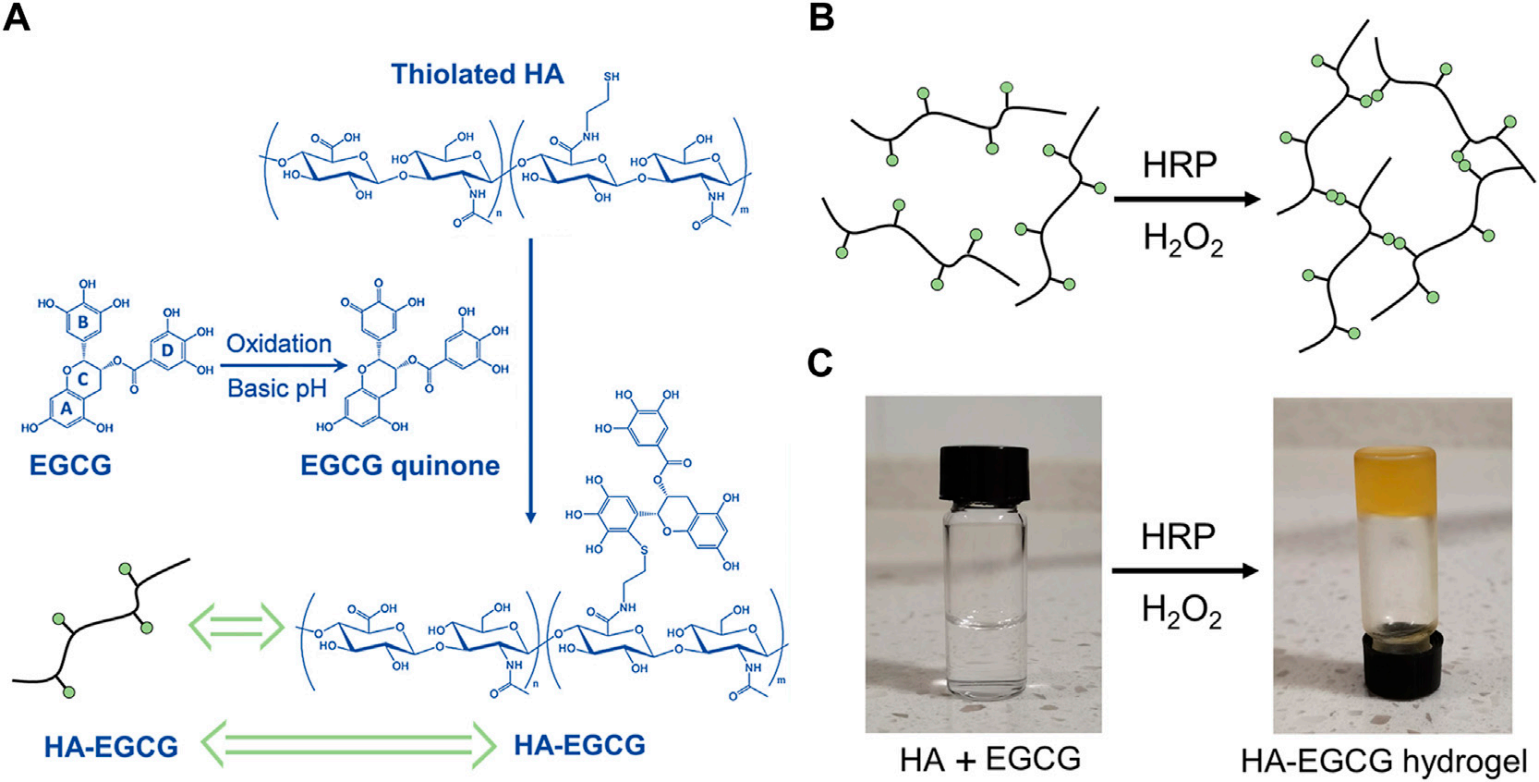
Preparation of HA-EGCG hydrogel. **(A)** Synthetic scheme of HA-EGCG conjugate. **(B)** Schematic diagram illustrating the formation of HA-EGCG hydrogel through oxidative coupling between two EGCG moieties catalyzed by HRP and H_2_O_2_. **(C)** Gross images of HA-EGCG conjugate solution (left) and HA-EGCG hydrogel (right) catalyzed by HRP and H_2_O_2_.

EGCG can undergo an autoxidation reaction to form an ortho-quinone in the pyrogallol part (B ring) at pH = 7.4. When excess EGCG mixed with thiolated HA, the nucleophilic addition of the thiol groups to the ortho-quinone and subsequent re-arrangement result in the conjugation of thiolated HA, specifically at the B ring in the EGCG (Cao et al., 2009). The preparation of HA-EGCG hydrogel is based on the previous well-established strategy (Lee et al., 2015; Liu et al., 2017). As illustrated in Figure 1A, the synthesis strategy of HA-EGCG conjugate was composed by multiple EGCG molecules grafted onto the HA backbone. The produced HA-EGCG conjugate was simple purification by dialysis against deionized water under nitrogen atmosphere. As shown in Figure 1B, the injectable hydrogel was prepared by using HA-EGCG conjugate *via* peroxidase catalyzed oxidative coupling reaction between EGCG moieties. After mixed by HRP and H_2_O_2_, the transparent HA-EGCG conjugate solution gradually formed yellow brown hydrogel within 1–2 min (Figure 1C). Previous studies have indicated that the catalytic oxidation of EGCG by HRP results in the production of phenoxy radicals, which can react with the other EGCG molecules to form EGCG dimers. In fact, the yellow brown color of HA-EGCG hydrogel suggested the existence of EGCG dimers (Zhu et al., 2000; Tanaka et al., 2009; Dai et al., 2020; Xu et al., 2021).

**FIGURE 2:**
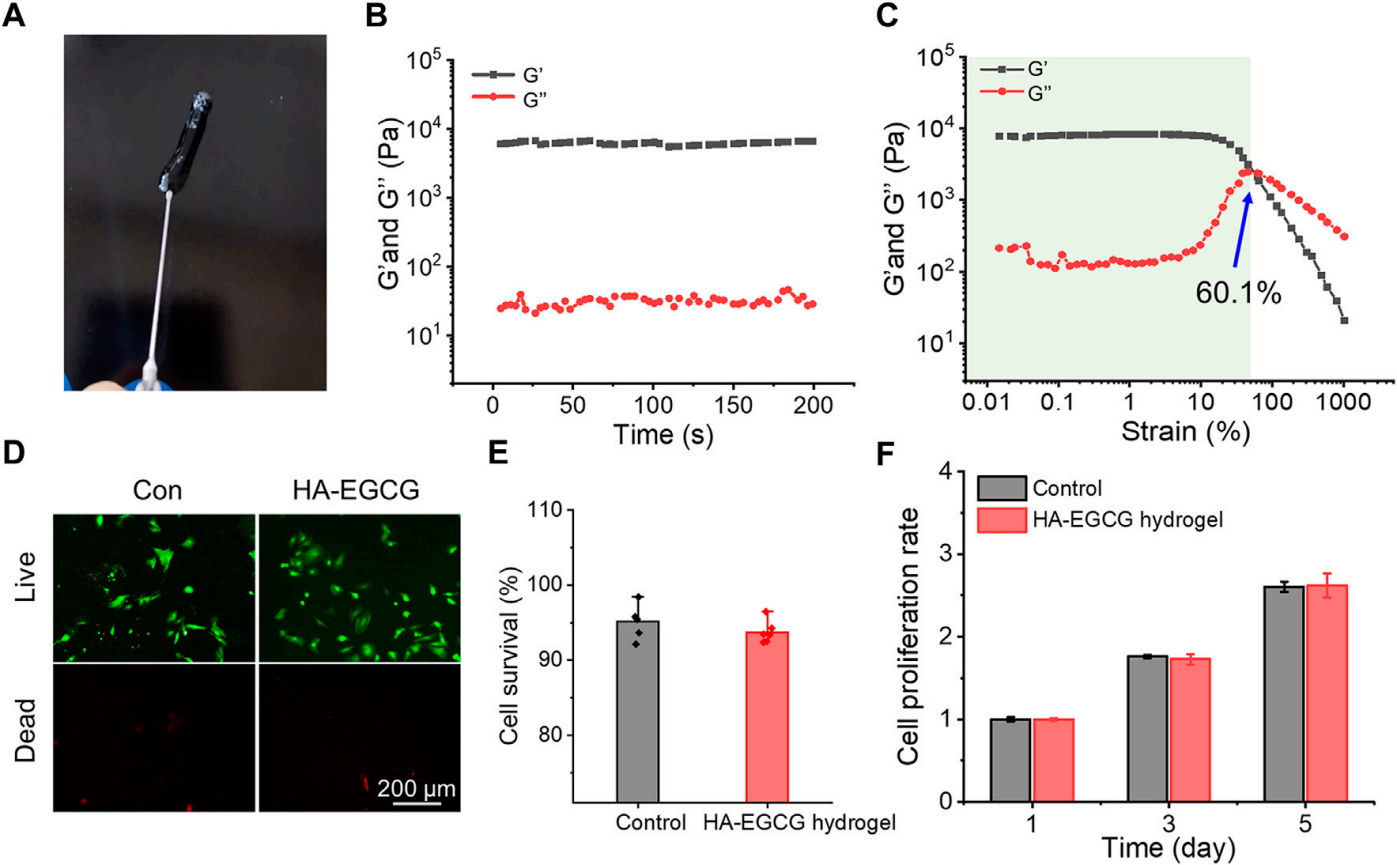
Characterization and biocompatibility of HA-EGCG hydrogel. **(A)** The injectability of HA-EGCG hydrogel extruding by a 26-gauge syringe. **(B)** Evolution of the G′ and G″ values over time for the HA-EGCG hydrogel. **(C)** Dependence of the G′ and G″ values over strain for the HA-EGCG hydrogel. **(D)** Calcein AM/PI staining of ADSCs. **(E)** Quantitative analysis of the survival rates of ADSCs (n = 6). **(F)** CCK-8 assay of ADSCs to detect cell proliferation (n = 3).

The injectability of hydrogel was depicted in Figure 2A, which can be easily pushed out by a 26-gauge syringe without clogging. This injectable ability of the HA-EGCG hydrogel provides the possibility of intra-articular injection as a cell carrier. As shown in Figure 2B, the value of G′ surpassed G″ and maintained constant in the time sweep mode suggesting the constructed polymer networks were stable. In addition, the representative strain amplitude sweep of hydrogel indicated that the values of G′ and G″ decreased with the strain range from 0.1% to 1000%, showing the typical viscoelastic behavior of hydrogels. In particular, the curves of G′ and G″ intersected at the strain of 60.1%, which was a crucial strain value for the breakdown of the polymer networks (Figure 2C). The viscosity of the hydrogels was tested by a viscometer. The result was revealed that the HA-EGCG hydrogel had high viscoelasticity, with viscosity index of 170 Pas and elasticity ratio of 51%, which is a favorable material for intra-articular injection.

As a carrier for stem cell delivery, the biocompatibility of the prepared hydrogel is crucial (Bai et al., 2020). As exhibited in Figure 2D, almost all the ADSCs seeded in the HA-EGCG hydrogel were stained with Calcein AM, indicating that they were living cells. As the control group for standardization, the survival rate of ADSCs seeded in the plates was 95.1 ± 2.19%, which was not significantly different from that cultured with the HA-EGCG hydrogel (93.7 ± 1.5%) (Figure 2E). In addition, the proliferation rates of ADSCs cultured in different substrates increased with the prolongation of culture time, and there was no significant difference between groups (Figure 2F). These outcomes proved that the HA-EGCG hydrogel has excellent biocompatibility and can well maintain the cellular activity of ADSCs encapsulated in it, thus making it eligible as candidates for cell delivery carrier.

### Degradation resistance of HA-EGCG hydrogel

**FIGURE 3:**
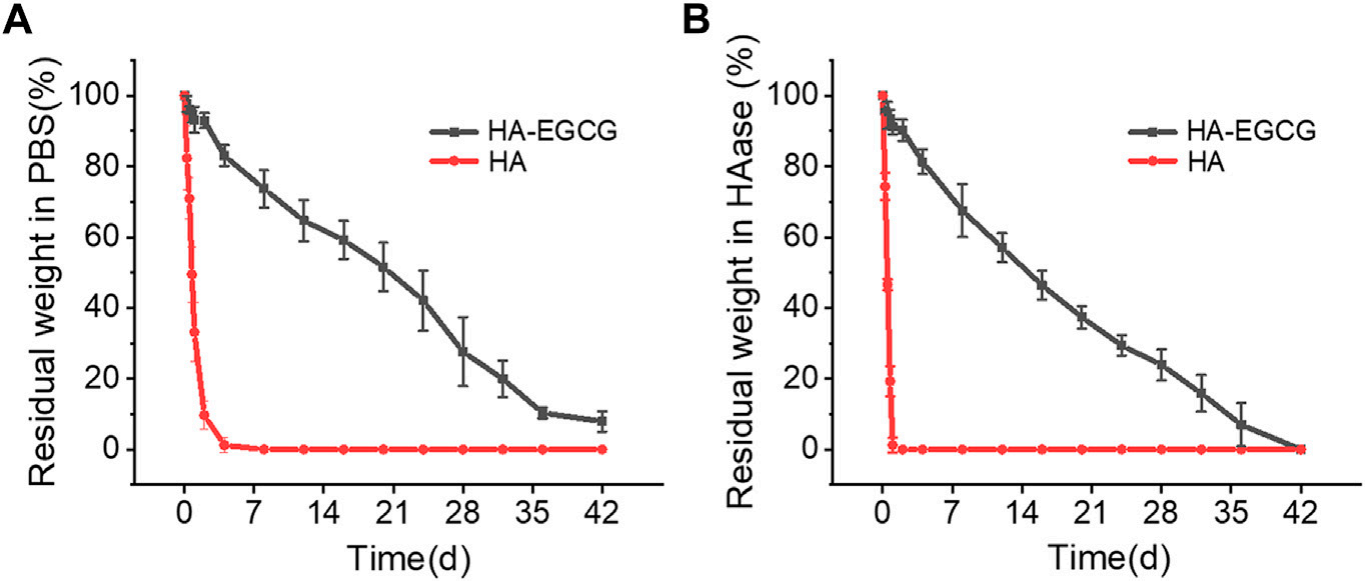
Degradation profiles of HA-EGCG hydrogel in PBS and HAase. **(A)** Degradation rate of the HA-EGCG hydrogel in PBS (n = 3). **(B)** Degradation of HA-EGCG hydrogel in PBS containing 15 units/mL HAase (n = 3).

HA with high viscoelasticity widely exists in joint synovial fluid, which plays an important role in shock absorption and lubrication (Donovan et al., 2022). Intra-articular injection of HA has been widely recommended in clinical practice and is considered a cost-effective treatment strategy for OA (Dai et al., 2020; Xu et al., 2021). However, the rapid degradation of HA in the articular cavity makes this administration strategy need to be repeated every week for several times. This repeated joint cavity puncture may bring some clinical complications, such as pain and joint cavity infection (Patil et al., 2022; Sax et al., 2022). To address these challenges, the HA-EGCG hydrogel with long-term degradation resistance was prepared by peroxidase catalyzed oxidative coupling process between EGCG moieties. As displayed in Figure 3A, the degradation time of HA-EGCG hydrogel in PBS was significantly prolonged, and there was still 51.57 ± 6.8% and 7.9 ± 2.9% residual amounts on the 20th and 42nd day. As a contrast, commercial HA almost completely degraded in PBS within 2 days.

In addition, in the presence of HAase (15 units/mL), the residual amounts of HA-EGCG hydrogel on the 20th was 37.3 ± 3.2%, and completely degraded on the 42nd day (Figure 3B). The results showed that HA-EGCG hydrogel exhibited significantly better resistance to HAase mediated hydrogel degradation than commercial HA. Specifically, commercial HA was completely degraded within 24 h, while about 91.2% of the HA-EGCG hydrogel still existed within the same time period. The HAase inhibitory capacity of HA-EGCG hydrogel may be conducive to improving the stability of its application in the joint cavity, thereby reducing the adverse effects of repeated administration.

### HA-EGCG hydrogel scavenging ROS and protecting cell viability under oxidative stress

**FIGURE 4:**
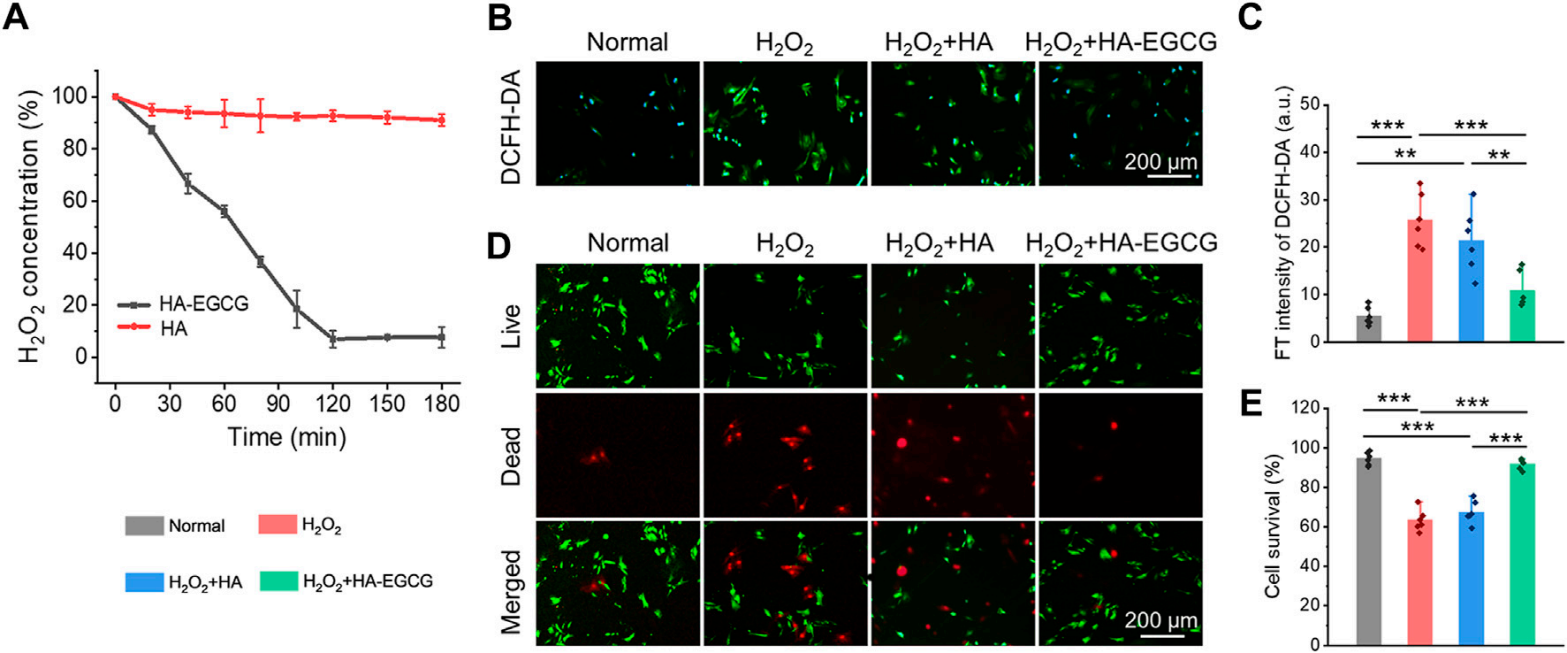
ROS-scavenging and cell viability protecting under oxidative stress. **(A)** Decomposition of H_2_O_2_ with HA-EGCG hydrogel and commercial HA (n = 3). **(B)** Intercellular ROS level validated by a ROS probe (DCFH-DA) after different treatments. **(C)** The quantitative analysis of fluorescence intensity of DCFH-DA to evaluate the ROS depletion. **(D)** Calcein AM/PI staining of ADSCs. **(E)** Quantitative analysis of the survival rates of ADSCs (n = 6) (*p < 0.05, **p < 0.01, and ***p < 0.001).

Considering the ROS accumulated microenvironment in the joint cavity of OA, hydrogel, as a cell delivery carrier, endowed with the ability of scavenging ROS, is a design strategy that needs to be included. As displayed in Figure 4A, commercial HA failed to show visible H_2_O_2_ scavenging capacity during the whole process. However, HA-EGCG conjugate endowed the hydrogel with excellent ROS-scavenging ability. Specifically, a time-dependent H_2_O_2_ scavenging efficacy detection assay exhibited that the HA-EGCG hydrogel decomposed approximately 46% of H_2_O_2_ in the first 60 min, and almost complete elimination was detected in the 120 min.

In addition, to imitate the oxidative stress microenvironment of OA *in vitro*, the ADSCs were cultured with 100 μM H_2_O_2_. The cells incubated under normal conditions without additional H_2_O_2_ introduced was set as the normal control group for comparisons. Cellular ROS eliminating ability of the HA-EGCG hydrogel was evaluated by a ROS-specific probe DCFH-DA (Figure 4B). As exhibited in Figure 4C, ADSCs in the H_2_O_2_+HA-EGCG group showed significantly lower green fluorescence intensity compared to the H_2_O_2_ group (*p* < 0.001) and H_2_O_2_+HA group (*p* < 0.01), indicating that the HA-EGCG hydrogel can subside the intracellular ROS efficiently to a normal standard like the Normal group. By continuously ROS-scavenging, a non-oxidative stress microenvironment was expected to construct, which was conducive to maintaining cell vitality. As depicted in Figure 4D, with the protection of HA-EGCG hydrogel, delivered ADSCs were almost green stained live cells under oxidative stress marked by Calcein AM/PI assay. Quantitative analysis displayed that the cell survival rates in Normal group, H_2_O_2_ group, H_2_O_2_+HA group, and H_2_O_2_+HA-EGCG group, were 94.5 ± 3.2%, 63.4 ± 5.5%, 67.5 ± 5.7%, and 92.0 ± 2.7%, respectively (Figure 4E). These results showed that the hydrogel with ROS-scavenging ability can well maintain the cell vitality of ADSCs under oxidative stress, which is expected to be used as a delivery carrier of stem cells to protect their functions in the joint cavity of OA.

### HA-EGCG hydrogel inhibited inflammatory cytokines expression and protected chondrogenic differentiation of ADSCs under oxidative stress

**FIGURE 5:**
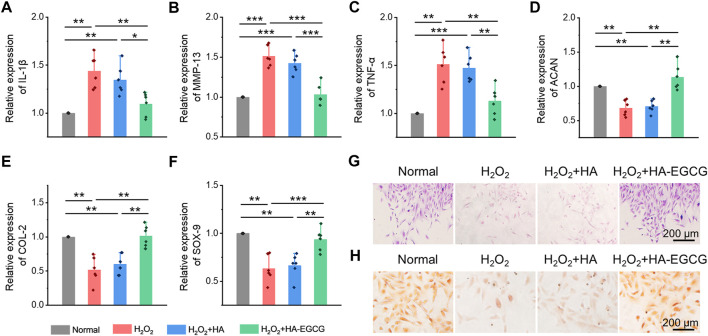
Inhibition inflammatory cytokines expression and induction chondrogenic differentiation of ADSCs under oxidative stress. **(A–C)** Real-time PCR analysis of inflammatory cytokines IL-1β, MMP-13, and TNF-α in ADSCs under oxidative stress (*n* = 6). **(D–F)** Real-time PCR analysis of chondrogenic-related genes ACAN, COL-2, and SOX-9 in ADSCs after chondrogenic induction (*n* = 6). **(G)** Alcain blue staining of ADSCs. **(H)** Safranine O staining of ADSCs (*p < 0.05, **p < 0.01, and ***p < 0.001).

Analysis has proved that H_2_O_2_ can induce cells to produce excessive ROS, which participates in many signal pathways by activating various cytokines to amplify inflammation reaction, impede cell viability, and prohibit chondrogenesis in OA (Hou et al., 2021). Herein, ADSCs cultured under oxidative stress were collected, and the expression of chondrogenic markers and inflammatory cytokines was detected by the real-time PCR. As showed in Figures 5A–C, when cells were treated with H_2_O_2_ (H_2_O_2_ group), the expression of inflammatory factors, including IL-1β, MMP-13, and TNF-α, increased significantly, while the addition of commercial HA (H_2_O_2_+HA group) cannot reverse this inflammatory state. However, co-cultured with HA-EGCG hydrogel (H_2_O_2_+HA-EGCG group), the level of inflammatory cytokines can be corrected and restored to a concentration roughly similar to that of the Normal group. Accordingly, this ROS-scavenging hydrogel alleviated the oxidative stress state and decreased the expression of inflammatory factors in cells, and ultimately facilitated the expression of chondrogenic related genes, such as ACAN, COL-2, and SOX-9 (Figures 5D–F). In addition, Alcain blue and Safranine O staining further verified that the HA-EGCG hydrogel was conducive to chondrogenic differentiation of ADSCs in the microenvironment of oxidative stress (Figures 5G, H). These results fully demonstrated that the HA-EGCG hydrogel inhibited the expression of inflammatory cytokines and induced cartilage differentiation of ADSCs under oxidative stress. It means that the HA-EGCG hydrogel as a protective carrier for cell delivery has a potential prospect in the treatment of OA.

### ROS-scavenging in synovial and cartilage tissues

**FIGURE 6:**
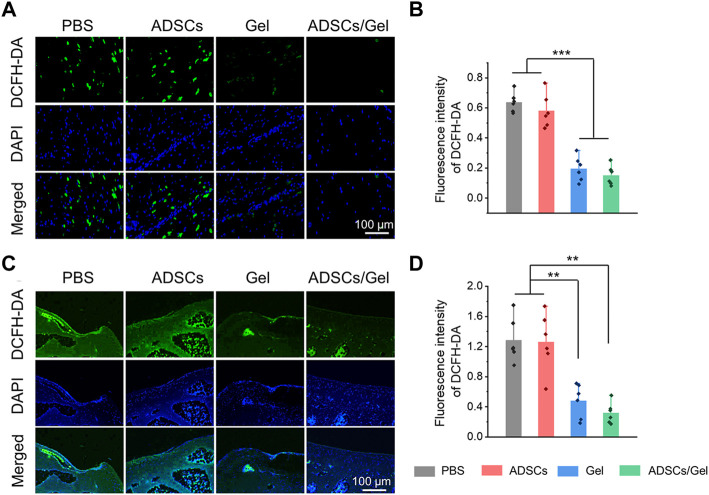
HA-EGCG hydrogel scavenging ROS in the joint cavity. **(A)** DCFD-DA labeling of ROS level in synovial tissues. **(B)** Quantitative analysis of the fluorescence intensity of DCFD-DA in synovial tissues (*n* = 6). **(C)** DCFD-DA labeling of ROS level in articular cartilage. **(D)** Quantitative analysis of the fluorescence intensity of DCFD-DA in articular cartilage (*n* = 6) (*p < 0.05, **p < 0.01, and ***p < 0.001).

The excessive oxidative stress caused by the imbalance between the production and elimination of ROS is regarded as a potential event to activate and accelerate pathological process of OA (Blanco et al., 2018). Previous study has rigorously shown that ROS will oxidize and subsequently destroy the homeostasis of cartilage, promote catabolism by enhancing chondrocyte apoptosis, and finally lead to excessive inflammation and degradation of cartilage matrix (Bolduc et al., 2019). Therefore, the researchers think that scavenging excessive ROS can improve the local microenvironment in the joint cavity and is a potential therapeutic strategy for OA. Herein, the ROS level in synovial tissues and articular cartilage tissues represents the severity of local oxidative stress, which is closely relevant to the progress of OA. At the 12th week after intra-articular injection therapy, we evaluated the ROS changes in synovial tissues by ROS probe (DCFH-DA), as exhibited in Figure 6A. The fluorescence intensity of the images indicated that intra-articular injection of HA-EGCG hydrogel or ADSCs incorporated HA-EGCG hydrogel can effectively reduce the ROS accumulation in the synovium of OA animals and relieve oxidative stress, compared with the PBS group and ADSCs group (Figure 6B). Furthermore, the level of ROS in articular cartilage also decreased significantly in the Gel group and ADSCs/Gel group (Figures 6C,D). These results proved that HA-EGCG hydrogel can remove ROS accumulated in the synovium and cartilage of OA, create a favorable microenvironment for transplanted stem cells, and thus starting the subsequent repair process effectively.

### HA-EGCG hydrogel induced synovial macrophage polarization and alleviated synovitis

OA is a chronic disease that involves all tissues of the joint and is featured with synovial inflammation, cartilage matrix progressive degeneration, subchondral osteosclerosis and exposure, and osteophyte formation (Zhang et al., 2022). Among them, synovial inflammation, as an initiating factor, is tied to the pathological aggravation of OA (Arra et al., 2020). Various evidences have indicated that the accumulation of ROS interacts with synovitis and worsens, which may show an important influence in the development of OA (Liu et al., 2022; Sunk et al., 2022). Inflammatory reaction exists in synovial tissues, that is, the severity of synovitis is associated with the symptoms of OA and the progress of disease to a large extent. A large number of macrophages with different activation states were observed in the synovium of OA patients at different stages (Yin J. et al., 2022; Jia et al., 2022). Among them, M1 phenotype macrophages are the main subgroups of inflammatory cytokines production, cartilage degradation, and osteophyte formation in the OA state, while M2 type macrophages have a protective effect on OA by secreting some anti-inflammatory factors (Lv et al., 2022; Wang et al., 2022). Previous studies demonstrated that inhibiting macrophages conversion to M1 type and promoting its polarization to M2 phenotype can alleviate the synovitis and delay the progress of OA (Zhang et al., 2018; Li D. et al., 2022; Wang and He, 2022). Therefore, regulating the polarization of synovial macrophages may be an alternative mean to manage OA.

**FIGURE 7:**
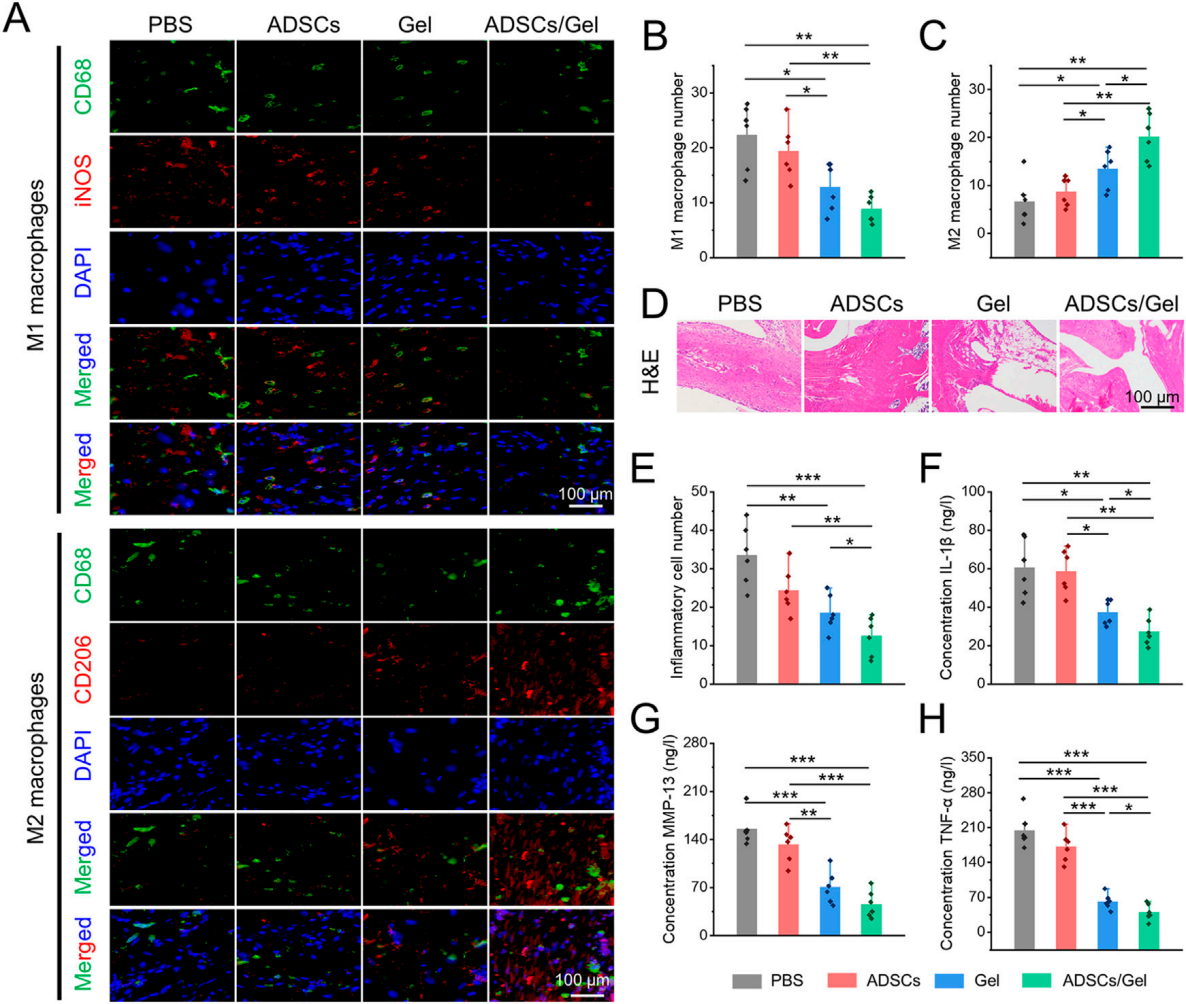
Intra-articular injection of ADSCs loaded HA-EGCG hydrogel induced synovial macrophages polarization and alleviated synovitis. **(A)** Immunofluorescence of CD68 plus iNOS to co-label M1 macrophages and CD68 plus CD163 to co-label M2 macrophages. **(B)** Quantitative analysis of the number of M1 macrophages per field (*n* = 6). **(C)** Quantitative analysis of the number of M2 macrophages per field (*n* = 6). **(D)** H&E staining of synovial tissues. **(E)** Quantitative analysis of the number of inflammatory cells infiltration in synovial tissues (*n* = 6). **(F–H)** The concentrations of various pro-inflammatory cytokines, including IL-1β, MMP-13, and TNF-α in synovial tissues by ELISA (*n* = 6) (*p < 0.05, **p < 0.01, and ***p < 0.001).

Herein, immunofluorescence double staining was carried out to label the polarization of synovial macrophages *in vivo* after intra-articular injection of therapeutic agents (Figure 7A **).** Specifically, M1 macrophages co-stained by CD68 plus iNOS in the Gel group and ADSCs/Gel group was prominently decreased compared with PBS group and ADSCs group (Figure 7B). As illustrated in Figure 7C, the number of M2 phenotype macrophages in synovium, which were labeled by CD68 plus CD163, in the PBS group, ADSCs group, Gel group, and ADSCs/Gel group were 6.7 ± 4.6, 8.7 ± 3.0, 13.5 ± 4.1, and 20.7 ± 5.0, respectively. In general, after intra-articular injection of HA-EGCG hydrogel, the M1 phenotype polarization of synovial macrophages in OA rats was significantly reduced, and the M2 phenotype polarization was significantly improved. It is worth noting that the HA-EGCG hydrogel loaded with ADSCs (ADSCs/Gel group) was more conducive to inducing the polarization of synovial macrophages to M2 type than the administration HA-EGCG hydrogel alone (Gel group), which may be attributed to the immune regulation of stem cells to enhance this polarization induction. Predictably, this effect of inducing pro-inflammatory macrophages to transform into an anti-inflammatory phenotype significantly inhibited synovial inflammation and inflammatory cell infiltration in each high-power field of the microscope through H&E staining in histology (Figures 7D,E).

In addition, the concentrations of various inflammatory cytokines (including IL-1β, MMP-13, and TNF-α) in synovial tissues were further evaluated by ELISA assays to confirm the severity of synovitis. As depicted in Figures 7F–H–H, these pro-inflammatory cytokines were decreased obviously in the Gel group and ADSCs/Gel group. In particular, the expression of IL-1β and TNF-α in the ADSCs/Gel group was markedly lower than that in the Gel group. In a word, these results indicated that intra-articular administration of HA-EGCG hydrogel or ADSCs loaded HA-EGCG hydrogel induced synovial macrophages polarization from M1 phenotype to M2, thus alleviating synovial inflammation and suppressing the concentrations of pro-inflammatory cytokines (including IL-1β, MMP-13, and TNF-α) in OA synovium. In general, these results have proved that the HA-EGCG hydrogel can remove the accumulated ROS and reduce the inflammatory state of synovium in OA joints, which reversed the adverse pathological microenvironment for ADSCs loaded in it, and provided great prospects for stem cells to repair damaged cartilage in the articular cavity.

### HA-EGCG hydrogel relieved cartilage destruction in OA

**FIGURE 8:**
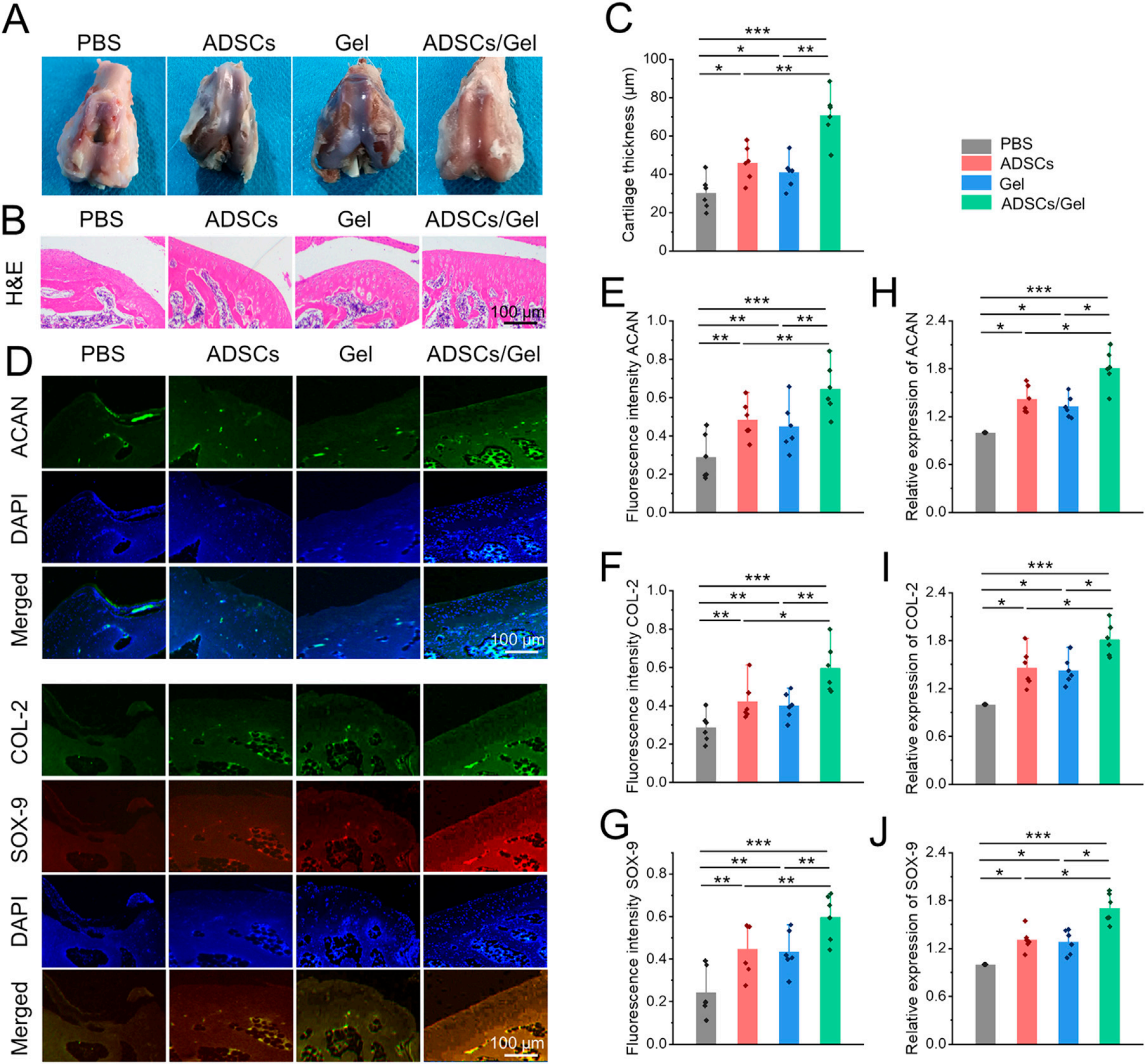
Intra-articular injection of ADSCs loaded HA-EGCG hydrogel repair the damaged cartilage in OA. **(A)** Gross observation of articular cartilages. **(B)** H&E staining of articular cartilage. **(C)** Quantitative analysis of the thickness of articular cartilage (*n* = 6). **(D)** Immunofluorescence of ACAN, COL-2, and COX-9 of the cartilage samples. **(E–G)** Quantitative analysis of fluorescence intensity of ACAN, COL-2, and COX-9 in the images (*n* = 6). **(H–J)** Real-time PCR detection the mRNA expression of ACAN, COL-2, and COX-9 in the cartilage samples (n = 6) (*p < 0.05, **p < 0.01, and ***p < 0.001).

The classic features of OA are erosion of cartilage surface, thinning of cartilage thickness, and subchondral bone exposure (Yue and Berman, 2022). With the great progress of cell therapy, intra-articular injection of stem cells has gradually become a new strategy to manage OA. Among them, MSCs are considered as a kind of alternative seed cells to treat OA because of their extensive sources, easy access, great potential for self-proliferation and multi-differentiation, and ability to regulate inflammation (Barry and Murphy, 2013; Bertoni et al., 2021; Hwang et al., 2021). Numerous previous clinical studies have systematically demonstrated that intra-articular injection of MSCs can improve cartilage damage and relieve pain in patients with OA (Aletto et al., 2022; Carvalho Schweich-Adami et al., 2022; Gunay et al., 2022). Specifically, ADSCs significantly reduced OA cartilage damage in rats, which can be achieved by down-regulating MMP-13 and COL-X and up-regulating COL-2. In addition, The paracrine action of ADSCs can show regulatory effects in chondrocyte proliferation, anabolism, and catabolism, as well as restore IL-1β Induced abnormal expression of molecular markers, including ACAN, COL-2, SOX-9, COL-X, SOX-6, IL-6, MMP-3, MMP-13, ADAMTS5, and ADAMTS9 in chondrocytes (Yan et al., 2022). However, after intra-articular delivering of MSCs, only a few cells adhere to the cartilage defects, their chondrogenic differentiation ability and paracrine action are not prominent in the harsh pathological microenvironment of joint cavity (Chang et al., 2016; Ha et al., 2019). Therefore, by regulating the ROS accumulation in the joint cavity to create a favorable microenvironment, it will contribute to the ADSCs to repair the OA articular cartilage effectively. Herein, HA-EGCG hydrogel as a cell protective carrier was used to incorporate ADSCs for intra-articular injection. The HA-EGCG hydrogel scavenged the accumulated ROS and alleviated progressive synovitis, thus improving the pathological microenvironment in OA joints. By regulating and improving the ROS accumulated microenvironment, the loaded ADSCs can maintain better cell vitality and chondrogenic differentiation potential, and it is expected to obtain better cartilage repair effect in OA. Gross observation of articular cartilages in the ADSCs/Gel group showed an intact appearance (Figure 8A). As showed in Figure 8B, H&E staining revealed that although the cartilage in the ADSCs group and Gel group showed an overall complete histomorphology, the thickness of the articular cartilage layer became thin and uneven, and the arrangement of deep layer chondrocytes was irregular and loose, even vacuolated. However, after intra-articular injection of ADSCs loaded HA-EGCG hydrogel, the histological morphology of cartilage was almost normal, and the thickness of cartilage layer was significantly higher than that of the PBS group (*p* < 0.001), ADSCs group (*p* < 0.01), and Gel group (*p* < 0.01), respectively (Figure 8C).

Subsequently, the chondrogenic markers in the articular cartilage were observed by immunofluorescence staining (Figure 8D). As exhibited in Figures 8E–G–G, the fluorescence intensity of ACAN, COL-2, and SOX-9 in the articular cartilage was obviously enhanced in the ADSCs/Gel group compared with the other three groups. In addition, at the gene level, the mRNA expression of ACAN, COL-2, and SOX-9 in the ADSCs/Gel group were also the highest among all experimental groups (Figures 8H,J–JFigures 8H,J). These results indicated that after the harsh microenvironment characterized by ROS accumulation in the OA articular cavity was improved, intra-articular injection of ADSCs loaded HA-EGCG hydrogel could maximize the role of enhancing anabolism of chondrocytes, and reducing the degradation of cartilage matrix, thereby greatly improving the pathological progress of OA.

## Conclusion

In this study, HA-EGCG hydrogel was prepared as a protective carrier of ADSCs for intra-articular injection in OA. The naturally derived injectable hydrogels with good biocompatibility and durable retention can capture the redundant ROS continuously and efficiently, thus protecting ADSCs from ROS-mediated death and bioactivity inhibition, including cell survival, proliferation and chondrogenic differentiation. Intra-articular administration of this ADSCs loaded hydrogel significantly induced synovial macrophages polarization to M2 phenotype and reduce synovial inflammation, thus maximizing the role of ADSCs on repairing cartilage destruction in OA, which providing a great potential for OA management.

## Data Availability

The original contributions presented in the study are included in the article/supplementary material, further inquiries can be directed to the corresponding author.
